# Surveillance of COVID-19 school outbreaks, Germany, March to August 2020

**DOI:** 10.2807/1560-7917.ES.2020.25.38.2001645

**Published:** 2020-09-24

**Authors:** Eveline Otte im Kampe, Ann-Sophie Lehfeld, Silke Buda, Udo Buchholz, Walter Haas

**Affiliations:** 1 Robert Koch Institute, Berlin, Germany

**Keywords:** SARS-CoV-2, COVID-19, school outbreaks, surveillance, Germany, children

## Abstract

Mitigation of the coronavirus disease (COVID-19) pandemic in Germany included school closures in early March 2020. After reopening in April, preventive measures were taken in schools. We analysed national surveillance system data on COVID-19 school outbreaks during different time periods. After reopening, smaller outbreaks (average: 2.2/week) occurred despite low incidence in the general population. School closures might have a detrimental effect on children and should be applied only cautiously and in combination with other measures.

As part of the containment activities for the coronavirus disease (COVID-19) pandemic, Germany’s federal states declared closure of primary and secondary schools on 16 March 2020. Within 3 days, schools in all federal states closed except for Saxony and Hesse where schools remained open for students who could not be cared for at home. However, no regular teaching was delivered. Limited reopening of secondary schools was approved on 20 April 2020. Primary schools offered reduced teaching hours only for final year students starting on 4 May 2020 and remained closed for other grades until the end of the summer break. After schools partially reopened, non-pharmaceutical interventions to reduce transmission were decided by each federal state individually [1]. From 22 June 2020, the summer break period started. As the date of the summer break varies from state to state, there was no time period after reopening when all schools were closed again in all states at the same time. 

Since closing schools is a severe disruption of children’s education [2] it is crucial to better understand the occurrence of school outbreaks during the pandemic as well as the impact of mitigation measures. The aim of our work was to describe COVID-19 school outbreaks in Germany during different periods of the pandemic to provide insights on the possible impact of school closures.

## Data source and management

We analysed data on mandatory notifications of laboratory-confirmed COVID-19 infections from the national surveillance system from 28 January 2020 until 31 August 2020. Laboratory confirmation requires detection of severe acute respiratory syndrome coronavirus 2 (SARS-CoV-2) nucleic acid by PCR or culture isolation of the pathogen. Physicians and laboratories notify the local public health authorities (PHA) who transfer data through the respective state PHA to the Robert Koch Institute (national public health institute) in Berlin. Notified COVID-19 cases are followed up by the local PHA for contract tracing, isolation, testing and, if applicable, outbreak investigation. All school outbreaks or outbreaks in other settings linked to a school outbreak were analysed if two or more cases were reported for one school outbreak. 

Since school education in Germany usually includes children 6 years and older, we excluded nine cases who were younger than 6 years, one case with unknown age and one outbreak that only had a case younger than 6 years and a 21-year-old case. We considered school outbreak cases up to 20 years of age as students. Except for vocational schools where students of different age groups can attend the same class, we assumed an age range of up to 2 years per school outbreak to represent same grades.

## School outbreaks in relation to school closures

Since the start of the COVID-19 pandemic and until 31 August 2020, 8,841 COVID-19 outbreaks comprising a total of 61,540 cases with documentation of the infection setting have been reported; 48 (0.5 %) of these outbreaks occurred in schools and included 216 cases. Almost half of the 216 cases occurred among persons 21 years and older (n = 102) followed by 45 cases among 11–14-year-old children, 39 cases among students aged 15–20 years and 30 cases among children aged 6–10 years.

Before schools were closed, school outbreaks were reported in every week, peaking in week 11 (six outbreaks) with a total of 30 of 216 cases and most cases reported in the age group 21 years and older (Figure). After all schools had been at least partially reopened for 1 week (week 20), outbreaks were reported in every week except for 2 weeks. The highest number of outbreaks (five) was reported in week 28, including 22 cases. Overall, the weekly number of outbreaks was lower during the period when the schools were partially open. The difference between the period before school closure and after reopening was small for the average number of outbreaks per week (Kruskal–Wallis p = 0.44) and the average number of cases per outbreak (Kruskal–Wallis p = 0.48). On average 2.2 outbreaks per week and four cases per outbreak were reported after schools reopened. Before school closures were implemented, an average of 3.3 outbreaks per week and six cases per outbreak were reported.

**Figure:**
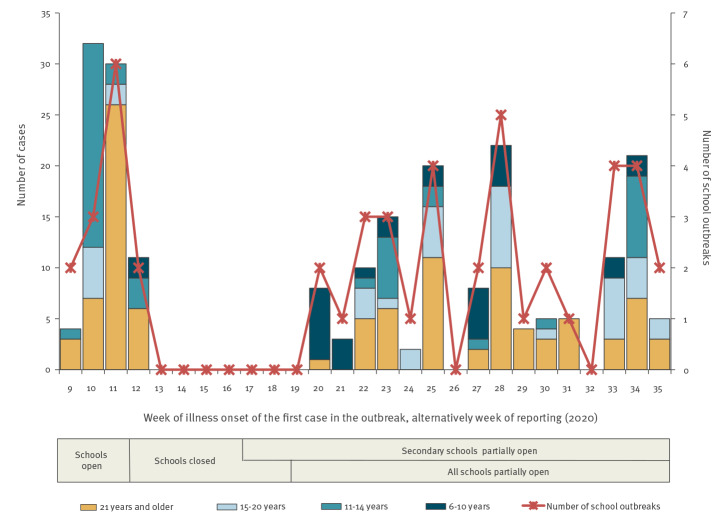
Number of school outbreaks (n = 48) and number of laboratory-confirmed COVID-19 cases (n = 216) by age and week of illness onset of the first case in the outbreak, Germany, 28 January–31 August 2020

COVID-19: coronavirus disease.

If data of illness onset were not available (n = 7), week of notification was used.

Data source: mandatory notifications of laboratory-confirmed COVID-19 infections from the German national surveillance system. Outbreaks that occurred during the observation period but reported after 31 August 2020 are not included.

## Frequency of symptoms 

Clinical data were available for 175 (81%) laboratory-confirmed COVID-19 cases associated with school outbreaks and for 18 of 30 in the youngest age group (Table 1). The proportion of cases with clinical data among the other age groups was similar. Among the 18 cases aged 6–10 years, data on symptoms compatible with COVID-19 were reported for only four. Local PHA reported symptoms suggestive of COVID-19, respectively, for 29 of 37 and for 80 of 90 cases with available clinical data in the group 11–14 years and 21 years and older, whereas such symptoms were reported for only 18 of 30 in the age group 15–20 years.

**Table 1:** Number and percentage of laboratory-confirmed COVID-19 cases for whom clinical data were collected with symptoms suggestive of COVID-19, overall and by age group, Germany, 28 January–31 August 2020 (n = 216)

	Total		Age groups (years)							
			6–10		11–14		15–20		≥ 21	
	n	%	n	%	n	%	n	%	n	%
Number of cases	216	100	30	100	45	100	39	100	102	100
No clinical data available^a^	41	19	12	40	8	18	9	23	12	12
Clinical data available^b^	175	81	18	60	37	82	30	77	90	88
Cases for whom clinical data were collected^b^ …	175	100	18	100	37	100	30	100	90	100
… without symptoms suggesting COVID-19	44	25	14	78	8	22	12	40	10	11
… with symptoms suggesting COVID-19	131	75	4	22	29	78	18	60	80	89

COVID-19: coronavirus disease.

^a^ Can include asymptomatic cases or cases for whom no clinical data were collected.

^b^ Can include asymptomatic or symptomatic cases with symptoms suggestive of COVID-19 or with symptoms suggestive of diseases other than COVID-19.

Data source: mandatory notifications of laboratory-confirmed COVID-19 infections from the national surveillance system.

## Age distribution and linkage to other settings

Five school outbreaks were linked to outbreaks in other settings (Table 2). Two school outbreaks in week 21 and 34 were each linked to an outbreak in a household and two school outbreaks were related to three household outbreaks each. Outbreak number 20 was connected to outbreaks in four different settings.

**Table 2:** Number and age of laboratory-confirmed COVID-19 cases, connection to outbreaks in other settings, by week of illness onset of the first case in a school outbreak^a^, Germany, 28 January–31 August 2020 (n = 216)

Outbreak^b^ number	Week of illness onset^a^	Number of cases reported	Age		Other setting linked to school outbreak
			< 21: in years (n)	n ≥ 21	
1	9	2	13 (1)	1	None
2	9	2	0	2	None
3	10	5	15 (1), 16 (1), 17 (1)	2	None
4	10	2	19 (2)	0	None
5	10	25	13 (13), 14 (7)	5	None
6	11	5	0	5	None
7	11	4	0	4	None
8	11^c^	11	20 (1)	10	None
9	11	3	13 (1), 14 (1)	1	None
10	11	2	0	2	None
11	11	5	17 (1)	4	None
12^d^	12	3	12 (2)	1	None
13^d^	12	8	8 (1), 10 (1), 12 (1)	5	None
School closure 16–18 March to 20 April 2020 (calendar weeks 12 to 17)					
14	20	4	7 (2), 8 (1), 10 (1)	0	None
15	20	4	8 (2), 10 (1)	1	None
16	21	3	10 (3)	0	Household
17	22	5	17 (1)	4	None
18	22	2	15 (1), 16 (1)	0	None
19	22	3	10 (1), 12 (1)	1	None
20	23	3	8 (1), 13 (1)	1	Household, accommodation, kindergarten, workplace
21	23	4	14 (1), 15 (1)	2	None
22	23	8	6 (1), 11 (1), 12 (1), 13 (2)	3	None
23	24	2	17 (1), 18 (1)	0	None
24	25	2	12 (1), 13 (1)	0	Household, household, household^e^
25	25	2	0	2	None
26	25^c^	9	17 (1), 18 (1), 20 (2)	5	None
27	25	7	6 (1), 10 (1), 17 (1)	4	None
28	27	2	10 (1), 11 (1)	0	Household, household, household
29	27	6	10 (4)	2	None
30	28	5	15 (1), 17 (1), 18 (1)	2	None
31	28	2	0	2	None
32	28	5	7 (1), 8 (1)	3	None
33	28	3	6 (1), 7 (1)	1	None
34	28^c^	7	16 (2), 17 (1), 18 (1), 19 (1)	2	None
35	29	4	0	4	None
36	30	2	0	2	None
37	30	3	11 (1), 15 (1)	1	None
38	31	5	0	5	None
39	33	2	17 (1), 18 (1)	0	None
40	33	2	10 (2)	0	None
41	33	5	15 (4)	1	None
42	33	2	0	2	None
43	34	9	12 (1), 13 (3), 17 (3), 19 (1)	1	Household^f^
44	34	8	12 (1), 13 (1)	6	None
45	34	2	7 (1), 8 (1)	0	None
46	34	2	11 (2)	0	None
47	35	3	17 (1)	2	None
48	35	2	19 (1)	1	None

COVID-19: coronavirus disease.

^a^ Alternatively by week of reporting (n = 7).

^b^ An outbreak was defined as at least two cases reported by a local public health authority for the same school.

^c^ Vocational school where students might attend teaching only on 2 days per week.

^d^ The reported day of illness onset of the first case was 16 March 2020 in both outbreaks.

^e^ Further events are associated with this outbreak.

^f^ This household outbreak had only one case. Reasons for an outbreak with one case could be contact tracing management or that secondary cases were attributed to another outbreak.

Data source: mandatory notifications of laboratory-confirmed COVID-19 infections from the national surveillance system.

For 10 of 48 school outbreaks, only cases in the age group 21 years or older were reported. In outbreaks that included cases younger than 21 years, the same grade was affected in 29 of the 48 outbreaks. Except for vocational schools, we observed two outbreaks affecting more than one grade during the period before school closure. After schools reopened partially, nine outbreaks included student cases from different grades. The largest number of cases per outbreak occurred in outbreak number 5 before any mitigation measures were implemented, with 20 cases in students aged 13 to 14 years and five cases among people 21 years or older.

## Discussion

By analysing data from Germany’s national surveillance system on laboratory-confirmed COVID-19 cases we could show that COVID-19 outbreaks in schools did occur. Most school outbreaks had few cases per outbreak, with more cases among older age groups who could have been staff or other persons epidemiologically linked to school outbreaks. In a minority of school outbreaks we could also find links to outbreaks in other settings, mostly within households, and our data suggest that mostly the same grades in a school were affected. In addition, albeit based on small numbers, we provided estimates of the proportion of symptomatic cases by age indicating that only a small proportion of primary school children were symptomatic.

Non-pharmaceutical interventions and hygiene measures applied after reopening of schools included opening schools for specific grades, staggering timetables, alternating between remote and on-site teaching, restricting class sizes, enhanced hand hygiene, wearing face masks, keeping distance between persons, ventilation of rooms as well as respiratory etiquette and policies for sick students and staff to stay at home [1]. When schools reopened, the incidence of COVID-19 in the general population was low and there was no community transmission [3]. Despite the low-incidence period and enhanced hygiene measures implemented in schools, school outbreaks occurred. The average number of outbreaks and of cases per outbreak was smaller after schools reopened than before school closure, suggesting that containment measures implemented in schools may have some protective effect. However, in some federal states, schools were closed again for summer break from June 2020 onwards, and our data show only weak evidence for a difference between the period before school closure and after reopening.

Our data were collected during outbreak investigations including testing of contacts. This allowed us to estimate the proportion of symptomatic infections among secondary cases, suggesting that four of 18 cases in children aged 6–10 years were asymptomatic. Our result is in agreement with available evidence that children with confirmed COVID-19 are less likely to be symptomatic than older age groups [4]. However, other studies reported asymptomatic proportions among children at around 20% [5-8]. One reason for this difference may be that the number of cases in this age group was small in our analysis and these studies. In addition, our students may have been presymptomatic during testing and for 12 of 30 6–10-year-olds, clinical data were not reported. It is possible that some of these may have been symptomatic cases. Overall, we estimated that 44 of 175 of the cases did not report any symptoms suggestive of a SARS-CoV-2 infection. This might be an underestimation as cases for whom no clinical data were available could have been asymptomatic.

There is some indication that transmission occurred within a school. As the number of student cases of the same grade was 25 in outbreak number 5, it is unlikely that no transmission occurred between students. Moreover, in some outbreaks, more than one grade was affected. However, considering class sizes of usually 20 to 25 students per class [9] the low number of cases in each age year suggests rather limited onward transmission within classes. In addition, the very small proportion of school outbreaks among all COVID-19 outbreaks in Germany suggests that schools have not been severely affected. This is in line with a report of COVID-19 school outbreaks in the European Union and European Economic Area region and the United Kingdom stating that only few COVID-19 school outbreaks have been documented [4]. On the other hand, a report from Israel on a major COVID-19 school outbreak indicated considerable SARS-CoV-2 transmission in a school after opening [10]. However, the class size in that school was larger (35–38 students per class) than average class sizes in Germany, and the Israeli outbreak coincided with a heat wave that may have negatively impacted on compliance with wearing face masks or other preventive measures.

There are some limitations to our analysis. Outbreaks, particularly in primary schools, may have been difficult to detect because the children may have been asymptomatic. On the other hand, if major onward transmission had occurred, larger outbreaks with spillover to older age groups would probably have been detected. Household outbreaks epidemiologically linked to schools are not always reported as linked outbreaks or as outbreaks at all. Moreover, we did not know in which class a student had been and can therefore not exclude that cases of similar age may had been in parallel classes. In addition, as the period of reopening schools coincided with relaxing measures in other settings, it is difficult to assess the impact of school reopening on transmission dynamics within a school. 

## Conclusion

Only few and mostly small COVID-19 school outbreaks had been reported in Germany overall, suggesting that the containment measures are sufficient to reduce spillover into the community.

While schools remain open, well-designed evaluations of the preventive measures are needed to assess effectiveness in terms of reducing SARS-CoV-2 transmission and to guide future decision-making during the COVID-19 pandemic. Moreover, school openings should be accompanied by developing surveillance capability and the ability to rapidly test, trace and isolate suspected COVID-19 cases and their contacts. To avoid detrimental effects on children, school closures should be applied only cautiously and in combination with other control measures. 
